# Common Bile Duct Dilatations in Asymptomatic Neonates: Incidence and Prognosis

**DOI:** 10.1155/2014/392562

**Published:** 2014-03-19

**Authors:** Shun-Feng Lin, Hung-Chang Lee, Chun-Yan Yeung, Chuen-Bin Jiang, Wai-Tao Chan

**Affiliations:** ^1^Department of Pediatrics, Chi Mei Medical Center, Liouying, No. 201 Taikang, Taikang Village, Liouying District, Tainan City 736, Taiwan; ^2^Department of Pediatrics, Mackay Memorial Hospital, No. 690 Section 2, Guangfu Road, East District, Hsinchu City 30071, Taiwan; ^3^Department of Pediatrics, Taipei Medical University, No. 250 Wu-Hsing Street, Taipei City 100, Taiwan; ^4^Department of Pediatrics, Mackay Memorial Hospital, No. 92 Section 2, Zhongshan North Road, Taipei City 10449, Taiwan

## Abstract

*Background*. This retrospective study reviewed 213 asymptomatic neonates with common bile duct (CBD) dilatations diagnosed via ultrasound to evaluate their incidence and outcomes. *Materials and Methods*. From August 2001 to July 2010, 18,230 abdominal ultrasound scans were performed as newborn screening. There were 213 (1.17%) cases of CBD dilatation. Dilatation of neonatal CBD was defined when its diameter was ≥2 mm. The neonates' birth history, CBD size, and follow-up results were analyzed. *Results*. In the 213 infants, four cystic dilatations (1.88%, 4/213) that were eventually diagnosed as choledochal cysts (CC). Among 209 neonates with fusiform dilatations (size 2.0–6.7 mm), 77 had ultrasound follow-up and 87% of them resolved spontaneously which were diagnosed as transient CBD dilatation (TCBDD). Eighty percent of TCBDDs resolved within 6 months. Patients with initial CBD size ≥3 mm had significantly lower resolution rate and neonates whose mothers are older than 35 years took longer time to resolve. *Conclusion*. The incidence of CBD dilatation in asymptomatic neonates was 1.17%. Eighty percent of TCBDDs resolved within 6 months. Regular ultrasound follow-up every 6 months may be appropriate for asymptomatic neonates with fusiform CBD dilatations to ensure resolution or progression.

## 1. Introduction

Dilatation of the common bile duct (CBD), also known as a choledochal cyst (CC), is one of the etiologies of conjugated hyperbilirubinemia, especially in children. Todani et al. classified CCs into five types [1, 2]. In children with cholestasis, CBD dilatation may be seen by ultrasound. Recently, the term common duct has been used in literature instead of CBD because the insertion of the cystic duct is not always identified clearly by ultrasound [3].

The accuracy of diagnosing CC via ultrasound is reported to be 71% if the CBD is ≥7 mm in diameter and 97% if ≥10 mm in diameter. Fusiform dilatation may be seen incidentally in secondary to biliary obstruction and in normal variants without clinical or laboratory abnormalities. Thus, if the CBD is <7 mm in diameter, the diagnosis of CC should be made carefully. Further investigation and follow-up should be done, especially in cases of fusiform dilatation [4, 5].

**Figure 1:**
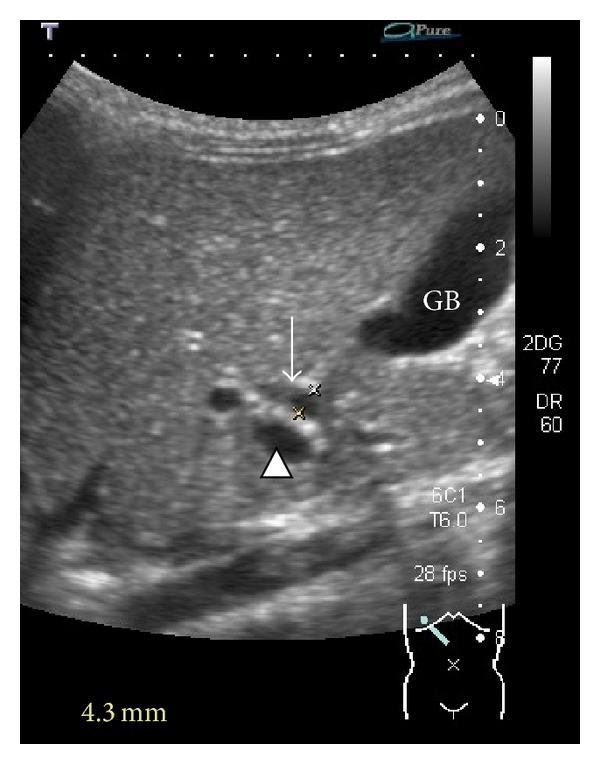
Common bile duct dilatation on ultrasound (arrow). The duct is measured as 4.3 mm. Portal vein (arrowhead). GB: gallbladder.

Cases of fusiform CBD dilatation <10 mm in diameter may be seen incidentally in ultrasound scans for other indications or as part of newborn screening (Figure 1). There is an interest in whether such cases will develop into CC or gradually resolve.

This retrospective study reviewed 213 cases of CBD dilatation in asymptomatic neonates diagnosed by abdominal ultrasound to analyze their incidence and outcomes.

## 2. Materials and Methods

Self-pay abdominal ultrasound, as a newborn screening test of the parents' own free will, has been performed since the year 2000 at Mackay Memorial Hospital. From August 2001 to July 2010, we did 18,230 neonatal abdominal ultrasound screen scans and found 213 CBD dilatations. Dilatation of neonatal CBD was defined when the CBD was ≥2 mm in diameter [4, 6–8]. If the CBD diameter was ≥10 mm, CC was highly suspected and regular ultrasound follow-up was performed monthly. Computed tomography (CT) or magnetic resonance cholangiopancreatography (MRCP) was done when the dilatations did not become smaller. Surgery was performed after CC was diagnosed.

If the CBD dilatation was <10 mm in diameter, follow-up ultrasound was arranged one month later and then every 3–6 months. Jaundice or other symptoms and signs of CC were also evaluated at the out-patient clinic. Resolution was defined when the CBD returned to <2 mm during infancy or <4 mm thereafter [6], without any clinical symptoms or signs. Cases that finally resolved were diagnosed as transient CBD dilatation (TCBDD).

A Toshiba system (30A, 50A, or 250; Toshiba, Tustin, CA) with a 3.5/5.0 linear or convex transducer and a Philips M2540A Envisor (Philips, Bothell, USA) with a 3.5/5.0 C8-5 curved array transducer were used. All operators were trained and experienced pediatric gastroenterologists. The neonates were fasted at least 3–4 hours before the examinations. Birth history, including the mother's age, gestational age, birth weight, sex, CBD size, and follow-up results, were recorded. This is a retrospective study and the hospital's Institutional Review Board approved the study.

Chi-square test, Student's *t-*test, Fisher's exact test, Mann-Whitney *U* test, log-rank test, and Kaplan-Meier survival curves were used, as appropriate, to compare the variables. All statistical analyses were performed using the SPSS for Windows version 17.0 (SPSS Inc., Chicago, IL, USA) software. Statistical significance was set at *P* < 0.05.

## 3. Results

**Figure 2:**
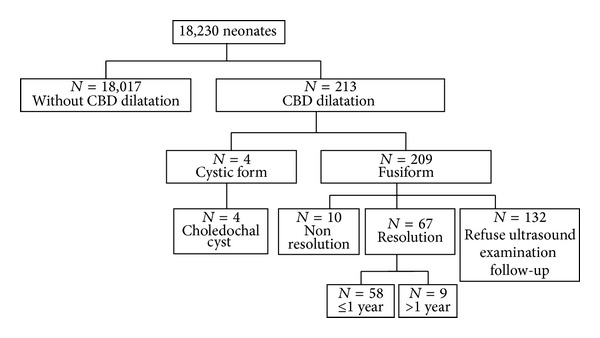
Overall initial and follow-up results of neonates who received abdominal ultrasound as newborn screening. CBD: common bile duct. *N*: number of neonates.

In the 18,230 neonates (9,533 males and 8,697 females) who underwent abdominal ultrasound scans, 213 (1.17%) had CBD dilatations. Among them, four cystic dilatations (1.88%, 4/213) were finally diagnosed as CC (size, 10.4–12.8 mm). The remaining 209 dilatations were fusiform (size 2.0–6.7 mm; mean diameter 2.6 ± 0.7 mm) (Figure 2). The mean mothers' age was 31.5 ± 4.2 years, mean gestational age was 272.1 ± 10.9 days, and mean birth weight of the neonates was 3114.4 ± 461.7 grams.

To assess the risk factors of fusiform CBD dilatations, the neonates were compared in terms of the mother's age, gestational age, birth weight, and sex (Table 1). There were no significant differences except in birth weight (*P* = 0.029) between neonates with and without fusiform CBD dilatations.

Among the 209 fusiform dilatations, 77 neonates had follow-up ultrasound scans, with a mean follow-up period of 156.0 ± 257.8 days and mean out-patient clinic follow-up period of 20.1 ± 23.8 months. The parents of the remaining 132 neonates refused ultrasound scans follow-up because they were asymptomatic. There were no significant differences in the general characteristics (gestational age, mother's age, birth weight, sex, and initial CBD size) between the two groups (Student's *t*-test).

**Figure 3:**
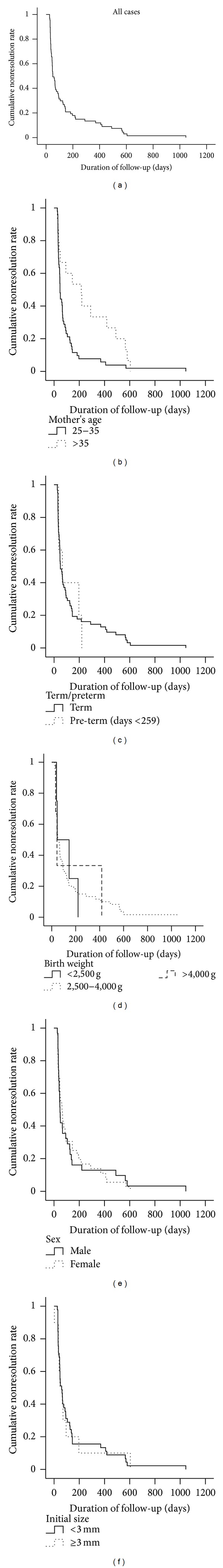
Kaplan-Meier survival curves for nonresolution rate, by log-rank test. (a) All cases. (b) Compared by mother's age (*P* = 0.013). (c) Compared by term and preterm status (*P* = 0.998). (d) Compared by birth weight (*P* = 0.988). (e) Compared by sex (*P* = 0.953). (f) Compared by initial CBD size (*P* = 0.466).

Sixty-seven fusiform CBD dilatations (87%) resolved gradually and were diagnosed as TCBDD. The remaining 10 dilatations did not resolve until the last evaluation (Figure 2). In the nonresolution group, CBD sizes were smaller than first scan in 8 neonates and the remaining two had CBD sizes increased (2.16 to 3.00 mm; 2.28 to 2.40 mm when followed up 134 days after first scan). In the resolution group, the median resolution time was 50 days (range 26–1,045 days, 95% confidence interval 34.0–66.0 days). Eighty percent of TCBDDs resolved within 6 months (Figure 3(a)). There was no significant difference between the resolution and nonresolution group as regards the mothers' age, birth weight, gestational age, and initial CBD size (Mann-Whitney *U* test).

The resolution rate was significantly lower when the initial CBD was ≥3 mm (*P* = 0.015, Fisher's exact test) (Table 2). The time to resolution was longer in mothers older than 35 years, based on the Kaplan-Meier survival curve (*P* = 0.013, log-rank test) (Figure 3).

Comparing neonates whose dilatations resolved within or beyond 1 year of age, there were no significant differences in gestational ages, birth weights, sex, initial CBD size, and mothers' ages (Mann-Whitney *U* test).

In the study population, there were 388 pairs of twins, one set of triplets, and 2,696 sets of siblings (5,542 neonates in total, including twins and triplets). Seven neonates with twins and 55 with siblings had fusiform CBD dilatations while their siblings did not. There was no significant difference in the incidence of fusiform CBD dilatations between singletons and twins/triplets.

## 4. Discussion

Common bile duct dilatations are not rare in Asian children and are more frequent in females [4, 9]. Choledochal cyst is a malformation of CBD and the pathogenesis is multifactorial [10–12]. The normal range of CBD size depends on age. Siegel stated that in infancy the normal CBD size should be <2 mm, <4 mm in childhood, and <7 mm after adolescence [6].

In this study CCs had an initial size of ≥10 mm and all fusiform dilatations were <7 mm. Although all CC neonates were female, there was no sex difference in those with fusiform CBD dilatations. This is consistent with a previous study, which suggested that CC might be considered if the CBD was ≥10 mm in diameter [4]. A previous study involving neonates with CC who needed surgery revealed that the cystic form had a higher incidence than the fusiform type and that this was associated with stenotic distal CBD [11].

The most common initial symptoms of CC in infants are abdominal mass and acholic stools. Older children may present with abdominal pain, while jaundice is seen in both infants and children [12]. In this study, all the infants were asymptomatic. The biliary dilatations were found via newborn screening sonographic tests.

With the availability of routine prenatal ultrasound screening, intra-abdominal cysts may be seen in antenatal scans as early as 15 weeks of gestation [13, 14]. The differential diagnosis of a right-sided intra-abdominal cystic mass includes duodenal atresia, hydronephrosis, ovarian cyst, and CC. Biliary atresia was also noted with the CCs, although the size of these CCs was smaller than that of isolated CCs [4, 15, 16]. Redkar et al. reported 13 cases with intra-abdominal cysts antenatally that were proven to be biliary atresia or CCs after surgery. The accuracy of CC diagnosis by antenatal sonogram scanning was 15%. All cysts were >10 mm in diameter when referred [17]. In the present study, only one neonate was diagnosed prenatally among the CC cases, and the size of the cyst was 12.8 mm by postnatal ultrasound scan.

The most common forms of CCs in children are type Ia, or cystic dilatation of the CBD, and type Ic, or the cylindrical or fusiform dilatation. Type Ic CC is considered if the CBD size is >10 mm, although fusiform CBD dilatation may be seen in normal variants and secondary to bile duct obstruction [4]. According to the study by Fitzpatrick et al., the incidence of CBD dilatation (the criterion they used was CBD size ≥1.2 mm) in cholestatic neonates was 9% [3]. In addition, they reported that CC was more likely and further interventions should be needed if the size of the CBD was greater than 4 mm. In contrast, if the CBD size was <3 mm, it was unlikely to be significant. Furthermore, they suggested that if the initial CBD size was 3–4 mm with no intrahepatic duct dilatation, ultrasound follow-up every 6 months should be done and full investigations should be arranged for any increase in size or if intrahepatic duct dilatation was seen [3].

In the current study, the incidence of CBD dilatation in asymptomatic neonates was 1.17% (including both fusiform dilatations and CCs). Of these cases, 87% of the fusiform CBD dilatations were TCBDDs, and 80% of TCBDDs resolved within 6 months, respectively. Even a CBD size as large as 6.7 mm resolved spontaneously (after 46 days). The pathophysiology of TCBDDs is not clear. The cases without resolution had follow-up ultrasound between 26 and 134 days after the first scans, and none had become symptomatic on their last follow-up. These cases might have resolved with longer follow-up.

The risk factors that predict the resolution of the fusiform CBD dilatations seemed to be the size of CBD and mother's age. Those with CBD size ≥3 mm had lower resolution rate, and those whose mothers are older than 35 years took a longer time to resolve.

The family occurrence of CBD dilatation has been discussed in previous studies. As many as 34 cases (17 pairs of siblings, twins, or parent and child) have been reported [18–20]. Genetic inheritance may be related to CBD dilatation. An X-linked dominant trait or an autosomal dominant trait has been suggested to be due to the female predominance. However, in some monozygotic twins, only one of the twins has been found to have CBD dilatation, so inheritance may just be one of the factors causing CBD dilatation. In the present study, there is no family occurrence in 388 pairs of twins, one set of triplets, and 2,696 sets of siblings, all of whom are newborns. However, previous studies have reported that the age of the affected families ranges between 7 days and 65 years [18–20].

Surgery is the only definitive management for CCs and may be necessary if progressive CBD dilatation, abnormal liver function, or obstructive jaundice is noted in children with CBD size <7 mm [4]. Recent studies suggest that early surgery is safer than delayed surgery and can prevent long-term complications. Laparoscopic surgery has become increasingly available in recent years and is considered safe compared to the traditional open surgery. For asymptomatic infants, the timing of surgery should be performed within 6 months of age, when the risk of anesthesia use is minimal, as long as the patient's clinical conditions permit [13, 21–28]. Diao et al. suggested that infants with asymptomatic CCs diagnosed prenatally should receive surgery within one month of age because liver fibrosis may develop immediately after birth [29].

This is a retrospective study and some data are not recorded in the charts, including parental history of CBD dilatation. Another bug of this study is refusing follow-up ultrasound scans in some cases that were still asymptomatic. Some risk factors may become significant if there are more follow-up cases. Future studies should include the cost effects of abdominal newborn screenings, more follow-up cases, and longer follow-ups for nonresolved cases to determine if some of them will become CCs.

## 5. Conclusion

Common bile duct dilatation has been detected by ultrasound in 1.17% of the asymptomatic neonates in this study, including 1.88% of the dilatations who had choledochal cysts with CBD size ≥10 mm. Of the fusiform dilatations, 87% resolved spontaneously. The resolution rate is lower in those CBD of size ≥3 mm and the time to resolution is longer if the mothers are older than 35 years. Because 80% of TCBDDs resolved within 6 months, we agree that regular ultrasound follow-up every 6 months may be appropriate for asymptomatic neonates with fusiform CBD dilatations to ensure resolution or progression.

## Figures and Tables

**Table 1:** Predictive factors of fusiform CBD dilatation*.

			Without dilatation	With dilatation	*P* value
			*n* = 18,017	*n* = 209	
Sex	Male	*n*	9,415	118	0.237
		%	98.80%	1.20%	
	Female	*n*	8,602	91	
		%	99.00%	1.00%	

			*n* = 17,747^†^	*n* = 207^††^	

Mother's age (years)	<25	*n*	883	6	0.334
		%	99.30%	0.70%	
	25–35	*n*	13,920	163	
		%	98.80%	1.20%	
	>35	*n*	2,944	38	
		%	98.70%	1.30%	

Term/preterm (<259 days)	Term	*n*	16,149	192	0.398
		%	98.80%	1.20%	
	Preterm	*n*	1,598	15	
		%	99.10%	0.90%	

Birth weight (grams)	<2,500	*n*	1,490	10	0.029
		%	99.30%	0.70%	
	2,500–4,000	*n*	15,854	188	
		%	98.80%	1.20%	
	>4,000	*n*	403	9	
		%	97.80%	2.20%	

*Chi-square test, excluding 4 neonates with choledochal cysts.

^†^270 neonates had incomplete birth history records.

^††^2 neonates had incomplete birth history records.

**Table 2:** Factors predicting resolution*.

			Nonresolution	Resolution	*P* value
			*n* = 10	*n* = 67	
Mother's age (years)	<25	*n*	1	0	0.113
		%	100.00%	0.00%	
	25–35	*n*	8	52	
		%	13.30%	86.70%	
	>35	*n*	1	15	
		%	6.30%	93.80%	

Term/preterm (<259 days)	Term	*n*	9	62	0.579
		%	12.70%	87.30%	
	Preterm	*n*	1	5	
		%	16.70%	83.30%	

Birth weight (grams)	<2,500	*n*	1	4	0.690
		%	20.00%	80.00%	
	2,500–4,000	*n*	9	60	
		%	13.00%	87.00%	
	>4,000	*n*	0	3	
		%	0.00%	100.00%	

Sex	Male	*n*	7	31	0.192
		%	18.40%	81.60%	
	Female	*n*	3	36	
		%	7.70%	92.30%	

Initial CBD size^†^ (mm)	<3	*n*	4	51	0.015
		%	7.30%	92.70%	
	≥3	*n*	6	13	
		%	31.60%	68.40%	

*Fisher's exact test.

^†^Three initial CBD sizes weresnot recorded in the “resolution” group.
